# Severe acute respiratory syndrome coronavirus 2 (SARS-CoV-2) seroprevalence: Navigating the absence of a gold standard

**DOI:** 10.1371/journal.pone.0257743

**Published:** 2021-09-23

**Authors:** Sahar Saeed, Sheila F. O’Brien, Kento Abe, Qi-Long Yi, Bhavisha Rathod, Jenny Wang, Mahya Fazel-Zarandi, Ashleigh Tuite, David Fisman, Heidi Wood, Karen Colwill, Anne-Claude Gingras, Steven J. Drews

**Affiliations:** 1 Epidemiology and Surveillance, Canadian Blood Services, Ottawa, Canada; 2 School of Epidemiology and Public Health, University of Ottawa, Ottawa, Canada; 3 Lunenfeld-Tanenbaum Research Institute at Mt. Sinai Hospital, Sinai Health System, Toronto, Canada; 4 Department of Molecular Genetics, University of Toronto, Toronto, Canada; 5 Dalla Lana School of Public Health, University of Toronto, Toronto, Canada; 6 National Microbiology Laboratory, Public Health Agency of Canada, Winnipeg, Canada; 7 Microbiology Department, Canadian Blood Services, Ottawa, Canada; 8 Division of Diagnostic and Applied Microbiology, Department of Laboratory Medicine & Pathology, University of Alberta, Edmonton, Canada; Waseda University: Waseda Daigaku, JAPAN

## Abstract

**Background:**

Severe acute respiratory syndrome coronavirus 2 (SARS-CoV-2) seroprevalence studies bridge the gap left from case detection, to estimate the true burden of the COVID-19 pandemic. While multiple anti-SARS-CoV-2 immunoassays are available, no gold standard exists.

**Methods:**

This serial cross-sectional study was conducted using plasma samples from 8999 healthy blood donors between April-September 2020. Each sample was tested by four assays: Abbott SARS-Cov-2 IgG assay, targeting nucleocapsid (Abbott-NP) and three in-house IgG ELISA assays (targeting spike glycoprotein, receptor binding domain, and nucleocapsid). Seroprevalence rates were compared using multiple composite reference standards and by a series of Bayesian Latent Class Models.

**Result:**

We found 13 unique diagnostic phenotypes; only 32 samples (0.4%) were positive by all assays. None of the individual assays resulted in seroprevalence increasing monotonically over time. In contrast, by using the results from all assays, the Bayesian Latent Class Model with informative priors predicted seroprevalence increased from 0.7% (95% credible interval (95% CrI); 0.4, 1.0%) in April/May to 0.7% (95% CrI 0.5, 1.1%) in June/July to 0.9% (95% CrI 0.5, 1.3) in August/September. Assay characteristics varied over time. Overall Spike had the highest sensitivity (93.5% (95% CrI 88.7, 97.3%), while the sensitivity of the Abbott-NP assay waned from 77.3% (95% CrI 58.7, 92.5%) in April/May to 64.4% (95% CrI 45.6, 83.0) by August/September.

**Discussion:**

Our results confirmed very low seroprevalence after the first wave in Canada. Given the dynamic nature of this pandemic, Bayesian Latent Class Models can be used to correct for imperfect test characteristics and waning IgG antibody signals.

## Introduction

Worldwide, more than 159 million people have been diagnosed with coronavirus disease 2019 (COVID-19), as of May 13, 2021 [1]. Yet, this is likely an underestimation of the true burden of severe acute respiratory syndrome coronavirus 2 (SARS-CoV-2) given testing is primarily used to confirm suspected infection as opposed to broad surveillance. For example, in Canada, testing was only accessible early in the pandemic to people who had symptoms, were known contacts of a case or had a relevant travel history [2]. This meant community transmission by asymptomatic or mildly symptomatic individuals was likely underestimated. Determining the proportion of individuals with evidence of an immune response to SARS-CoV-2 can provide a more comprehensive assessment of prevalence to assist public health officials in making policy decisions. This prompted an urgent need for seroprevalence studies and accurate anti-SARS-CoV-2 immunoassays to estimate the true burden of disease.

While multiple commercial and in-house immunoassays to detect anti-SARS-CoV-2 antibodies are available, to date no gold standard exists [3]. Furthermore, laboratorians have described multiple examples of discordance between assays [4]. Some of this variability is in part due to the assays which vary significantly by the isotype (i.e. IgA, IgM, IgG), viral antigens (i.e. spike or nucleocapsid protein and whether full-length or partial), and test performance (i.e. sensitivity/specificity). It is also known that anti-SARS-COV-2 antibodies wane over time which can further affect the sensitivity and specificity of the assays [5–7]. Additionally, biological differences between individuals can lead to different antibody profiles. Given these overlapping challenges of estimating seroprevalence, relying on a single assay (regardless which assay this may be) may bias results.

In the absence of a gold standard, using results from multiple assays may improve accuracy. However, which methods are appropriate for estimating SARS-CoV-2 seroprevalence has not been defined. One method is to use a composite reference standard (CRS); a traditional approach used in clinical settings based on prespecified rules based on results from multiple assays [8]. More recently, Bayesian Latent Class Analysis (BLCA) has become more mainstream in diagnostic studies [9]. In contrast to CRS which classifies individuals as either positive or negative, BLCA uses a likelihood-based approach from multiple imperfect assays to estimate test characteristics and prevalence. Given the uncertainty of the assay performance, we evaluated multiple methodological approaches to estimate SARS-COV-2 seroprevalence during the first COVID-19 wave in Canada using four unique assays.

## Methods

### Study design and population sampling

We conducted a serial cross-sectional study among blood donors in Canada between April and September 2020 (prior to COVID-19 vaccine availability). Canadian Blood Services (CBS) collects approximately 850,000 blood donations per year from a combination of fixed and mobile sites in all larger cities and most urban areas from all provinces in Canada except Quebec [10]. Blood donors (>17 years old) must meet numerous selection criteria to ensure that they are in good health and at low risk of infectious disease. Beginning in March 2020, donors were deferred for two weeks if they were diagnosed with SARS-CoV-2 infection or if they were in contact with a known case. Each month 1500 deidentified samples were randomly selected by collection site by region, age and sex to be reflective of the donor population across Canada. Data on the collection site, birth year, sex and Forward Sortation Area (FSA) of the residential postal code for each donor were extracted. The Research Ethics Board of the Canadian Blood Services and Lunenfeld-Tanenbaum Research Institute (LTRI) (REB study #20-0194-E) approved this study and exempted study-specific consent.

### SARS-CoV-2 antibody testing

Retention EDTA plasma samples were aliquoted and frozen at -20°C at the CBS laboratory in Ottawa. Each sample was tested for SARS-CoV-2 IgG antibodies using four assays. The Abbott Architect SARS-Cov-2 IgG assay which targets the nucleocapsid antigen (Abbott-NP), (Abbott, Chicago IL) and three in-house IgG ELISA chemiluminescent assays recognizing distinct recombinant viral antigens: full length spike glycoprotein (Spike), spike glycoprotein receptor binding domain (RBD), and nucleocapsid (NP), were tested at the CBS laboratory in Ottawa and the Gingras laboratory [11, 12] at the LTRI in Toronto, respectively. Table 1 summarizes each antibody assay by: platform, antigen targets and how reactivity was determined.

**Table 1:** Assay characteristics.

Assay	Assay platform	Capture Antigen (IgG)	Manufacture	Cut-offs (positive)	Cut-off reference
Abbott-NP	Chemiluminescent microparticle immunoassay	Nucleocapsid	Abbott	≥1.40	Manufacture
Spike	Chemiluminescent ELISA	spike	Gingras Lab	≥0.190	3 SD + negative mean^a^
RBD	Chemiluminescent ELISA	RBD	Gingras Lab	≥0.186	3 SD + negative mean^a^
NP	Chemiluminescent ELISA	Nucleocapsid	Gingras Lab	≥0.396	3 SD + negative mean^a^

Abbott-NP, Abbott Architect SARS-Cov-2 IgG assay targeting nucleocapsid antigen; Spike, full length spike glycoprotein; RBD, spike glycoprotein receptor binding domain; NP, nucleocapsid.

^a^3 Standard deviations (SD) + negative mean is a standard approach to choosing cut-off thresholds for ELISA based assays. Briefly, the relative ratio values of the negative controls from 20–22 different tests were used. Ratios are transformed on the log10-scale and then mean 3 SD determines the cutoff. Values are then exponentiated to identify cut off listed in the table.

### Analysis

We evaluated the correlation between the individual assays by kappa statistics. In the absence of a gold standard, we examined multiple approaches to estimate seroprevalence. First, seroprevalence was estimated by individual assays based on pre-defined thresholds (Table 1). Then we used a series of composite reference standards to identify a “true” positive if a sample was reactive by a combination of two or more assays. Finally, we estimated seroprevalence using Bayesian Latent Class Models (BLCM).

### Bayesian latent class analysis

In this study the “latent” unobservable target was evidence of SARS-CoV-2 infection (based on IgG positivity). Instead of relying on one imperfect assay, this iterative model leverages the data from multiple imperfect assays to estimate the “true” prevalence and test characteristics [13, 14]. Given any one of four assays could assign an individual to be positive or negative, there was a maximum of 16 (2^4^), possible diagnostic phenotypes. We assumed each assay was independent of the others, conditional on the individual’s unknown antibody status. This means that the probability of obtaining a given diagnostic phenotype depended on the probability that an individual had been truly infected with SARS-CoV-2 and on the outcome of each assay given the underlying exposure status. Briefly, we estimated parameters in a Bayesian framework using a Gibbs sampler to produce Markov chain Monte Carlo (MCMC) simulations. We ran 50,000 iterations, with the first 5000 steps discarded as burn-in [15]. Given the uncertainty of assay performance in this donor population, we compared goodness of fit parameters using informative, weakly informative and non-informative priors. Informative priors were based on the manufactures assumed sensitivity assumed specificity (S1 Table). Expert opinion defined weakly informative priors (sensitivity ranging from 60%-100% and specificity from 90%-100%, for each assay). We assumed an uniformed distribution for the model using non-informative priors. We verified convergence of all MCMC chains. We reported posterior means and 95% credible intervals (CrI) for all estimated parameters overall and by two-month intervals using SAS (version 9.1, SAS Institute, Cary, NC). For more additional details on Bayesian Latent Class analysis please refer to Cheung et al, 2021 [16].

## Results

Between April and September 2020, a total of 8999 healthy blood samples were assessed for SARS-CoV-2 antibodies by four distinct assays. Most donors (96%) were between 20–69 years old, there were slightly more male donors (52%) compared to female donors (48%) and there was representation from all provinces across Canada except Quebec. Donor characteristics remained consistent over the study period (S2 Table).

### Individual assays

**Fig 1:**
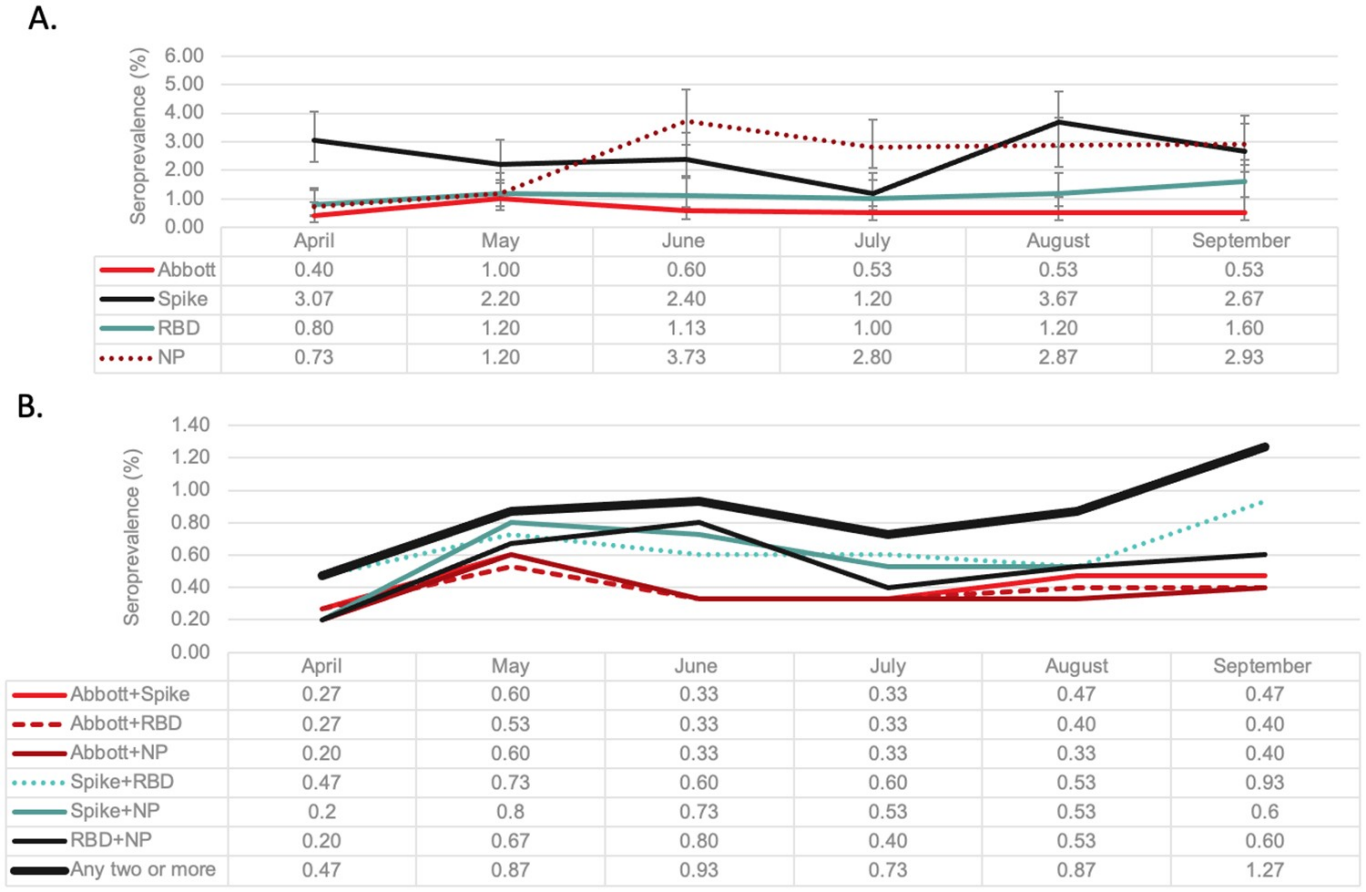
**A**. Seroprevalence by month over the first COVID-19 wave in Canada by individual assays. Each line represents seroprevalence rates (summarized in table below) monthly between April and September 2020 (during the first COVID-19 Wave) based thresholds for each assay. Abbott Architect SARS-Cov-2 IgG assay (Abbott-NP) and three in-house IgG ELISA assays recognizing distinct recombinant viral antigens: full length spike glycoprotein (Spike), spike glycoprotein receptor binding domain (RBD), and nucleocapsid (NP). **B**. Seroprevalence by month over the first COVID-19 wave in Canada by various composite reference standards (results from four anti-SARS-CoV-2 immunoassays). Each line represents seroprevalence rates based on predefined definitions. CRS based on a combination of reactive samples using Abbott-NP, Spike, RBD and NP. Positivity based on “any two or more” was determined by a reactive sample from two or more assays. Since we are not comparing CRS, we did not include 95% CI for each data point (all overlapping).

We evaluated seroprevalence rates over time by the individual assays (Fig 1a). Overall, there was significant variability by the assays over time. The Abbott-NP assay consistently remained lower than the ELISA-based assays. Seroprevalence based on the spike assay was 3.1% in May, dropped to 1.2% in July and then plateaued around 3%. Rates were lower and more stable by RBD that started at 0.8% and increased to 1.6% by September. In contrast the NP assay increased significantly from May (1.2%) until June (3.7%). The signal to cut off ratios remained relatively stable over time for all assays (S1 Fig). Overall, the correlation between the assays was low (kappa score, 0.28 (95% CI 0.21, 0.34)). Given concurrent negative results the percent agreement was highest between Abbott-NP and RBD (kappa 0.43 (95% CI 0.33, 0.51).

### Composite reference standards

Given screening occurred in a low prevalence setting, to minimize false positive results, we assumed a true positive was more likely when two or more assays were positive. *A priori*, relying on two pre-specified assays resulted in a range of seroprevalence estimates that ranged from 0.2% to 0.5% in April to 0.4% to 1.4% in September (Fig 1b). Any two assays (from four) resulted in a seroprevalence that increased significantly over time from 0.5% (95% CI 0.3%, 1.1%) in April to 1.3% (95% CI 0.8, 2.0) in September (p = 0.02) (Fig 1b).

### Bayesian latent class model

**Fig 2:**
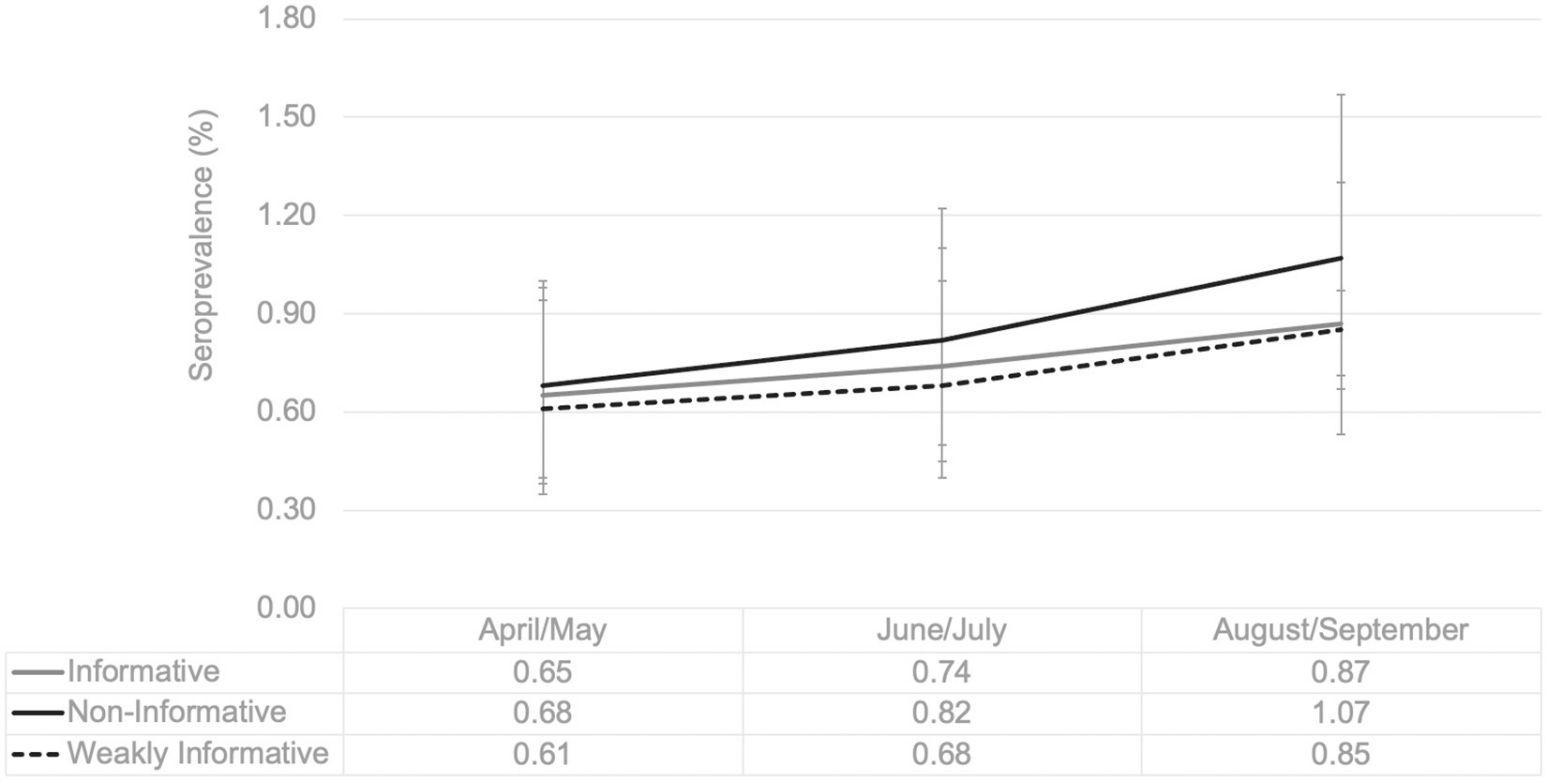
Seroprevalence estimates by different BLCM (informative, weakly informative and non-informative priors). Each line represents seroprevalence rates (summarized in table below) derived from posterior means of three BLCMs (comparing informative, weakly informative and non-informative priors) bi-monthly between April and September 2020 (during the first COVID-19 Wave). Error bars represent 95% Credible Intervals.

From 16 possible diagnostic phenotypes, 13 were observed among the 8999 sampled (eight phenotypes had at least 10 observations). The most frequent profile was “all negative” (95.0% (95% CI 94.6, 95.5) followed by only positive by the individual ELISA-based assays (Spike only (1.8%, 95% CI 1.5, 2.0) and NP only (1.7, 95% CI 1.5, 2.1). Only 32 samples were positive for all four assays (0.4, 95% CI 0.3, 0.5) (Table 2). Overall seroprevalence was estimated to be 0.8% (95% CrI 0.6, 1.0%); 0.8% (95% CrI 0.6, 1.0%); 0.8% (95% CrI 0.7, 1.0%) using informative, weakly informative and non-informative priors, respectively. Fig 2 illustrates temporal trends in seroprevalence by BLCA comparing the various models. The model with the non-informative prior consistently was higher than the other two models, but the difference was not statistically significant. Given the uncertainty of test characteristics, we compared the observed vs predicted values of the three BLCM and found the informative model had the best model fit and identified the “all negative” phenotype most accurately (S3 Table).

**Table 2:** Diagnostic phenotypes of anti-SARS-CoV-2 immunoassay results from 8999 samples tested.

	Commercial	In-house ELISA			Observed	
	Abbott-NP	Spike	RBD	NP	Number	% (95% CI)
	(n = 54)	(n = 228)	(n = 104)	(n = 214)		
NONE	-	-	-	-	8552	95.0 (94.6, 95.5)
ALL	+	+	+	+	32	0.4 (0.3, 0.5)
All—Abbott-NP	-	+	+	+	9	0.1 (0.1, 0.2)
NP Only	-	-	-	+	156	1.7 (1.5, 2.1)
RBD Only	-	-	+	-	39	0.4 (0.3, 0.6)
Spike Only	-	+	-	-	158	1.8 (1.5, 2.0)
Spike + RBD	-	+	+	-	15	0.2 (0.1, 0.3)
RBD + NP	-	-	+	+	7	0.1 (0.0, 0.3)
Abbott—NP Only	+	-	-	-	17	0.2 (0.1, 0.3)
Spike + Abbott-NP	+	+	-	-	2	0.0 (0.0, 0.1)
Spike + NP	-	+	-	+	9	0.1 (0.1, 0.2)
All–NP	+	+	+	-	2	0.0 (0.0, 0.1)
All–RBD	+	+	-	+	1	0.0 (0.0, 0.1)

Abbott-NP, Abbott Architect SARS-Cov-2 IgG assay targeting nucleocapsid antigen; Spike, full length spike glycoprotein; RBD, spike glycoprotein receptor binding domain; NP, nucleocapsid.

The test characteristics (sensitivity and specificity) varied significantly by the different assays (Table 3). Overall, the ELISA based assays had higher sensitivity than the Abbott-NP. Abbott-NP had a sensitivity of 58.5% (95% CrI 46.3, 70.6%) and a specificity of 99.8% (95% CrI 99.7, 99.9%). RBD had the highest specificity (99.5% (95% CrI 99.3, 99.7%)) and NP had the lowest specificity (98.2% (95% CrI 97.9, 98.4%)). Negative predictive values of all assays were very high (ranging from 99.4% to 99.8%). The Abbott-NP had the highest positive predictive value at 87.5% (95% CrI 81.3, 93.8%) while the ELISA based assays that ranged from 21.0 to 59.8%. The ELISA-based assays did not significantly wane over the first six months of the pandemic, the test characteristics of the Abbott assay varied more (Table 3). Sensitivity Abbott-NP assay waned the most from 77.3% (95%CrI 58.7, 92.5) in April/May to 64.4% (95% CrI 45.6, 83.0) in August/September. Similar trends were observed using weakly informative and non-informative priors (S4 Table).

**Table 3:** Seroprevalence and assay characteristics overall and bi-monthly based on the Bayesian latent class analysis with informative priors.

	Overall				April/May		June/July		August/September	
Seroprevalence	0.76% (95% CrI 0.58, 0.97)				0.65% (95% CrI 0.38, 0.98)		0.74% (95% CrI 0.45, 1.11)		0.87% (95% CrI 0.53, 1.29%)	
	Sensitivity	PPV	Specificity	NPV	Sensitivity	Specificity	Sensitivity	Specificity	Sensitivity	Specificity
Spike	93.5%	27.7%	98.1%	99.8%	95.0%	98.0%	93.6%	98.8%	93.6%	97.6%
	(88.7, 97.3)	(23.2, 33.7)	(97.9, 98.4)	(99.6, 100.0)	(90.1, 98.2)	(97.4, 98.4)	(88.0, 97.6)	(98.4, 99.2)	(88.0, 97.6)	(97.0, 98.1)
RBD	89.1%	59.8%	99.5%	99.8%	89.3%	99.5%	89.2%	99.6%	88.7%	99.3%
	(84.1, 93.5)	(50.0, 71.4)	(99.3, 99.7)	(99.6, 99.9)	(83.7, 93.8)	(99.3, 99.8)	(98.4, 99.2)	(83.6, 93.9)	(83.0, 93.4)	(99.0, 99.6)
NP	78.8%	21.0%	98.2%	99.6%	79.9%	99.5%	80.5%	97.3%	78.5%	97.6%
	(74.1, 83.2)	(17.2, 25.3)	(97.9, 98.4)	(99.3, 99.7)	(74.9, 84.5)	(99.2, 99.7)	(75.6, 84.9)	(96.7, 97.9)	(73.5, 83.3)	(97.1, 98.1)
Abbott-NP	58.5%	87.5%	99.8%	99.4%	77.3%	99.7%	60.2%	99.8%	64.4%	99.9%
	(46.3, 70.6)	(81.3, 93.8)	(99.7, 99.9)	(99.1, 99.6)	(58.7, 92.5)	(99.5, 99.9)	(41.2, 78.5)	(99.5, 99.9)	(45.6, 83.0)	(99.8, 100.0)

CrI, Creditable Interval; PPV, Positive Predictive Value; NPV, Negative Predictive Value; Abbott-NP, Abbott Architect SARS-Cov-2 IgG assay targeting nucleocapsid antigen; Spike, full length spike glycoprotein; RBD, spike glycoprotein receptor binding domain; NP, nucleocapsid.

**Fig 3:**
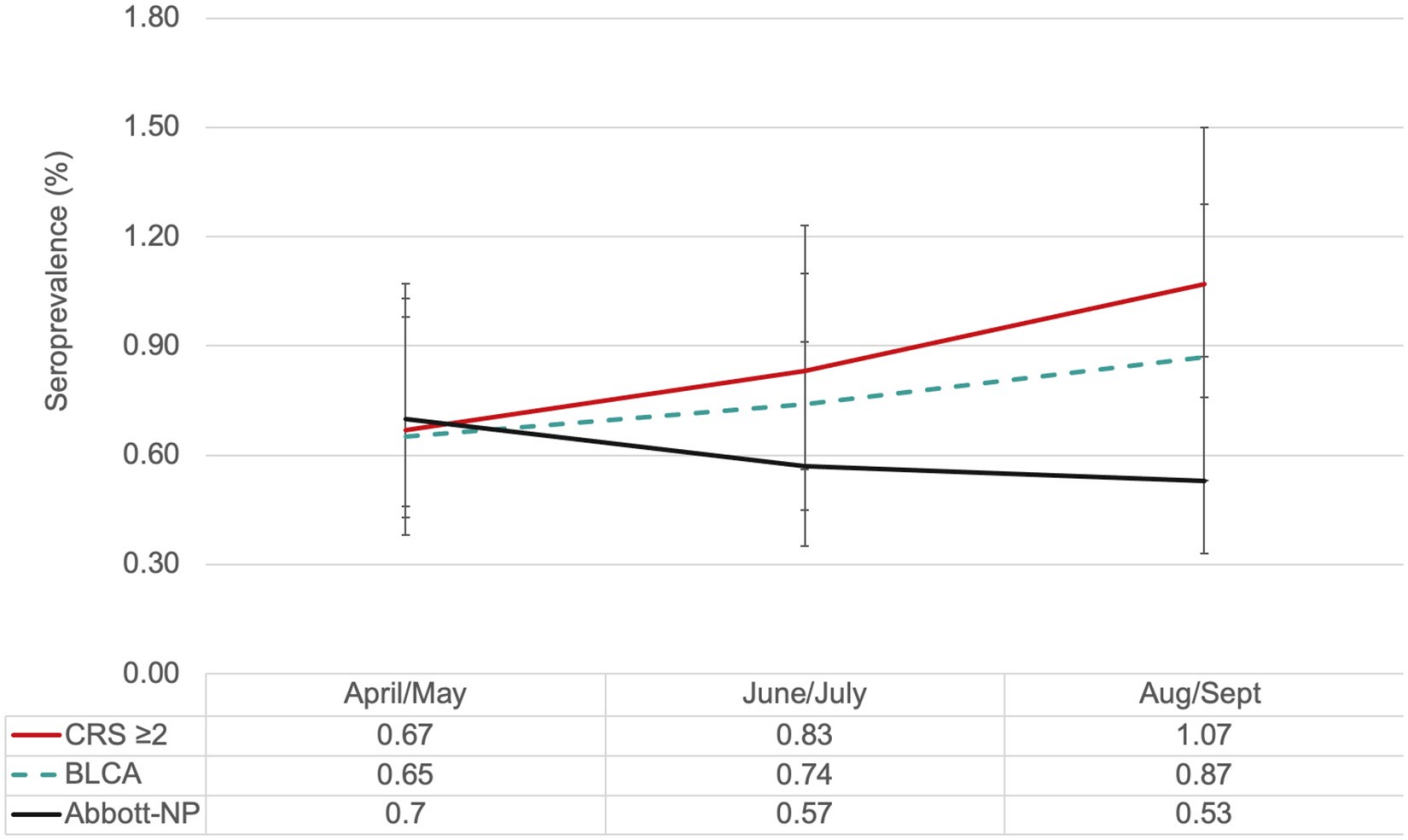
Summary comparison of seroprevalence rates by analytical methods. Each line represents seroprevalence rates (summarized in table below) derived from four analytical methods bi-monthly between April and September 2020 (during the first COVID-19 Wave). > = 2 proteins (positivity was determined by a reactive sample from two or more assays), BLCA-Bayesian latent class analysis with informative priors, results are posterior means and error bars are 95% CrI and Abbott-NP is a single commercial assay.

Overall the latent class model and CRS (using the rule > = 2 reactive assays out of four) yielded similar results and there was no evidence of waning seroprevalence rates over time (Fig 3). While the Abbott-NP (a common commercial assay) did wane over time.

## Discussion

In the absence of a gold standard, we evaluated multiple assays and methodological approaches to estimate SARS-CoV-2 seroprevalence in healthy Canadian blood donors. None of the individual assays resulted in seroprevalence increasing monotonically over time. Seroprevalence estimates were similar by either BLCM or a composite reference standard when at least two positive assays (out of four) were used to determine a “true” result. However, by using the BLCM, we were able to derive time-updated test characteristics that could be used to adjust for waning antibody signals.

Gaps in laboratory testing during the first wave, the significant proportion of asymptomatic or pauci-symptomatic infections, as well as a continuing pandemic have prompted public health authorities in Canada to continue to invest in serological surveys to evaluate the true burden of SARS-CoV-2. Yet unique biological and epidemiological challenges exist when estimating seroprevalence, particularly in low prevalence settings. We recently conducted a scoping review and identified 33 seroprevalence studies among blood donors worldwide. From the 33 studies, 27 unique assay combinations were identified, more than half of studies used a single assay to determine prevalence and less than a third accounted for imperfect test performance [17]. Results from this study suggest relying on a single assay to determine prevalence in a low prevalence setting may significantly bias results.

The variability in the number of diagnostic phenotypes may be associated with the interindividual variability of the immune response. SARS-CoV-2 infects cells using a spike glycoprotein to bind to human angiotensin-converting enzyme 2 (ACE2) [18–20]. The receptor binding domain attached to spike mediates both viral binding and fusion events and all proteins are targets for neutralizing monoclonal antibodies [21, 22]. Biologically, it is not clear why a person may differentially express antibodies against SARS-CoV-2, but among our sample, we found 13 distinct diagnostic phenotypes. The discordance between assays may also be a product of imperfect test characteristics. In this study, we used one commercial assay for which the manufacture originally reported a sensitivity of 95.9% and specificity of 99.6%. Later real-world reports suggested the sensitivity was as low as 92.7% [23–28]. Results from this study suggest significantly lower sensitivity. While it is customary to assume that assay performance remains static, amid this dynamic pandemic, waning antibody signals may compromise correct classification of prior SARS-CoV-2 exposure. We have previously shown in a longitudinal study that the NP signal in the ELISA-based assay wanes faster than spike or RBD [12]. Consistent with previous reports, we found the nucleocapsid signal from the Abbott assay also wanes faster than spike or RBD [29, 30]. This suggests that NP-based assays may be identifying more recent exposures.

It should be noted that waning antibody signals do not necessarily mean waning cellular mediated immunity. Indeed, recent studies suggest in the absence of detectable antibody signals there is evidence of neutralization associated with longer lasting immunity [31, 32]. Therefore, without adjusting for waning antibody signals we may be underestimating SARS-CoV-2 seroprevalence. At this point in time, it remains unknown what the true measures or correlates of immunity are in the Canadian population. The data presented here does not address whether some blood donors may have mounted a cellular immune response with an antibody response that waned by the time of serologic testing. We also note that the presence of antibodies does not imply that those antibodies are neutralizing; although we have assessed for spike and RBD antibodies, we have not attempted to understand the neutralizing capacity of these donor specimens against wild type strains of SARS-CoV-2 or emerging variants in Canada. In the next steps of our analysis we will be undertaking studies to understand the neutralizing capacity of these donor specimens to SARS-CoV-2.

Our study has several strengths. First this study is nested within a large national seroprevalence survey which to date has tested >179,000 samples using the Abbott-NP assay since the beginning of the pandemic in Canada. While we tested only a fraction of the samples, the sample demographic and seroprevalence rates (based on Abbott-NP) were very similar, illustrating the generalizability of our results nationally [33].

Given the uncertainty around the assay characteristics specifically among a donor population, we used multiple methodological approaches to estimate seroprevalence and report all findings. One of the strengths of the BLCA is the ability to estimate assay performance in the absence of a gold standard. Given limited resources, it may not be feasible to evaluate seroprevalence using four unique assays. However, smaller nested studies with more comprehensive antibody data within larger surveys can be used to correct for measurement errors. For example, we used the sensitivity and specificity of the BCLA from this study to adjust for national seroprevalence estimates recently published between May-July 2020 [33]. We reported seroprevalence was 0.74% (95%CI 0.68, 0.80), but after reanalyzing the data with updated sensitivity/specificity, based on the BLCM with informative priors, we found that the corrected seroprevalence was 27% higher at 0.94% (95% CI 0.83, 1.05%). As the pandemic continues, the proportion of recent and older infections will continue to vary over time and having an ability to correct these time varying assay characteristics will become even more important.

Our study also has weaknesses. This study was conducted among blood donors, based on selection criteria to be allowed to donate blood donors may be healthier than the general population [34]. However, a recent study compared seroprevalence estimates from European blood donors to household surveys targeting the general population and found seroprevalence rates to be very similar [35]. In this analysis we assumed the assays were conditionally independent, meaning there were no jointly false-positive or false-negative results between the assays. We evaluated this assumption by assessing changes in sensitivity and specificity of each assay after leaving one assay out. Although we found insignificant changes in test characteristics (S5 Table), it is possible this assumption does not hold, potentially biasing results. Future studies are planned to explore different correlation structures. Assay performance is based on predefined thresholds. For the Abbott assay we used the manufacturer’s ≥1.4 cut off, but recent reports do suggest reducing the threshold to >0.8 to increase sensitivity and to account for waning antibody signals. However, the sensitivity and specificity was not provided by the manufacture to evaluate this alternative threshold. All four assays only probed for IgG meaning that we did not measure IgM and IgA, which may provide some neutralizing capacity in some individuals as anti-SARS-CoV-2 IgG titers begin to rise. In other donors, different profiles of anti-SARS-COV-2 IgM, IgA and IgG may also provide different profiles of humoral protection. Finally, we did not assess for the SARS-CoV-2 neutralizing capacity of donor specimens nor the avidity of the IgG antibody responses in those donors.

## Conclusions

We used multiple analytical methods and assays to confirm very low seroprevalence (~1%) among a healthy population of Canadian blood donors after the first COVID-19 wave [36]. We also found antibody signals by all the assays waned over time and this impacted seroprevalence rates. These findings suggest significant limitations to using a single assay to estimate SARS-CoV-2 seroprevalence in a low prevalence setting. We recommend that seroprevalence studies use multiple assays on either their entire sample or a representative subset to estimate seroprevalence more accurately in the future. As seroprevalence studies enter a new era of tracking natural and vaccine induced humoral immunity, highly sensitive methods will continue to be needed to adjust for waning antibodies and imperfect test characteristics.

## Supporting information

S1 Fig Signal to cut off ratio (S/Co) by calendar month April (4) until September (9). Red lines represent thresholds. Abbott-NP (1.4) (n = 54 positive) based on the manufacture’s recommendations. Spike (0.190) (n = 228 positive); RBD (0.186) (n = 104 positive); and NP (0.396) (n = 214 positive). Abbott-NP, Abbott Architect SARS-Cov-2 IgG assay targeting nucleocapsid antigen; Spike, full length spike glycoprotein; RBD, spike glycoprotein receptor binding domain; NP, nucleocapsid.
(DOCX)

S1 Table Informative priors. ^1^Sensitivity and Specificity are based on manufactures, given the uncertainty the range was based on expert opinion. Abbott-NP, Abbott Architect SARS-Cov-2 IgG assay targeting nucleocapsid antigen; Spike, full length spike glycoprotein; RBD, spike glycoprotein receptor binding domain; NP, nucleocapsid.
(DOCX)

S2 Table Baseline characteristics of blood donor populations by month. (DOCX)

S3 Table Goodness of fit (comparing modelled results (expected) to observed data). Abbott-NP, Abbott Architect SARS-Cov-2 IgG assay targeting nucleocapsid antigen; Spike, full length spike glycoprotein; RBD, spike glycoprotein receptor binding domain; NP, nucleocapsid.
(DOCX)

S4 Table **A.** Assay Characteristics Overall and Bi-monthly based on the Bayesian Latent Class Analysis with Non-Informative Priors. PPV, positive predictive value; NPV, negative predictive value; Abbott-NP, Abbott Architect SARS-Cov-2 IgG assay targeting nucleocapsid antigen; Spike, full length spike glycoprotein; RBD, spike glycoprotein receptor binding domain; NP, nucleocapsid. **B.** Assay Characteristics Overall and Bi-monthly based on the Bayesian Latent Class Analysis with Weakly-Informative Priors. PPV, positive predictive value; NPV, negative predictive value; Abbott-NP, Abbott Architect SARS-Cov-2 IgG assay targeting nucleocapsid antigen; Spike, full length spike glycoprotein; RBD, spike glycoprotein receptor binding domain; NP, nucleocapsid.
(DOCX)

S5 Table Overall sensitivity and specificity after leaving an assay out (informative priors). Abbott-NP, Abbott Architect SARS-Cov-2 IgG assay targeting nucleocapsid antigen; Spike, full length spike glycoprotein; RBD, spike glycoprotein receptor binding domain; NP, nucleocapsid.
(DOCX)

S1 Data (XLS)

S1 Code (DOCX)
