# Supplementary figures and images for: Severe acute respiratory syndrome coronavirus 2 (SARS-CoV-2) seroprevalence: Navigating the absence of a gold standard
Source: PLoS One. 2021 Sep 23;16(9):e0257743. doi: 10.1371/journal.pone.0257743 (PMC8459951; doi:10.1371/journal.pone.0257743)

**S1 Fig.**


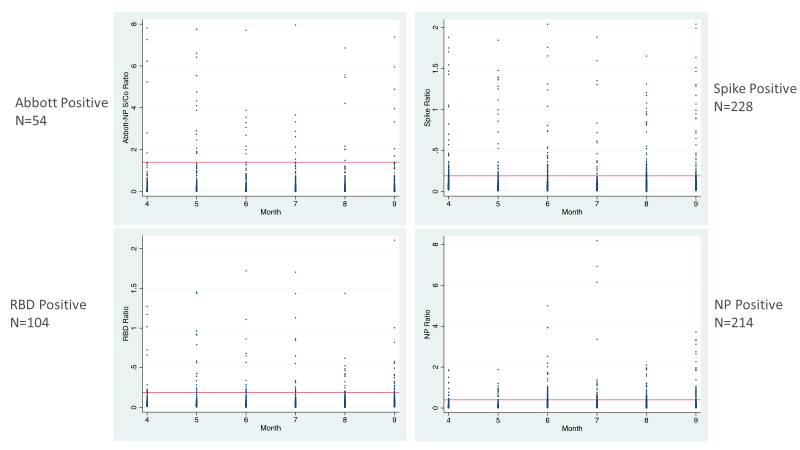

Supplement: S1 Fig — Red lines represent thresholds. Abbott-NP (1.4) (n = 54 positive) based on the manufacture’s recommendations. Spike (0.190) (n = 228 positive); RBD (0.186) (n = 104 positive); and NP (0.396) (n = 214 positive). Abbott-NP, Abbott Architect SARS-Cov-2 IgG assay targeting nucleocapsid antigen; Spike, full length spike glycoprotein; RBD, spike glycoprotein receptor binding domain; NP, nucleocapsid. (DOCX) [file pone.0257743.s001.docx]
