# Supplementary material for: Severe acute respiratory syndrome coronavirus 2 (SARS-CoV-2) seroprevalence: Navigating the absence of a gold standard
Source: PLoS One. 2021 Sep 23;16(9):e0257743. doi: 10.1371/journal.pone.0257743 (PMC8459951; doi:10.1371/journal.pone.0257743)
Supplement: S1 Table — 1Sensitivity and Specificity are based on manufactures, given the uncertainty the range was based on expert opinion. Abbott-NP, Abbott Architect SARS-Cov-2 IgG assay targeting nucleocapsid antigen; Spike, full length spike glycoprotein; RBD, spike glycoprotein receptor binding domain; NP, nucleocapsid. (DOCX) [file pone.0257743.s002.docx]

**S1 Table.**

|  | **Sensitivity** | **Range^1^** | **Specificity** | **Range^1^** |
| --- | --- | --- | --- | --- |
| **Spike** | **94%** | **(89- 99%)** | **98%** | **(93-100%)** |
| **RBD** | **89%** | **(84-95%)** | **99%** | **(95-100%)** |
| **NP** | **80%** | **(75-85%)** | **99%** | **(94-100%)** |
| **Abbott** | **92%** | **(60-95%)** | **95%** | **(94-100%)** |
