# Supplementary material for: Severe acute respiratory syndrome coronavirus 2 (SARS-CoV-2) seroprevalence: Navigating the absence of a gold standard
Source: PLoS One. 2021 Sep 23;16(9):e0257743. doi: 10.1371/journal.pone.0257743 (PMC8459951; doi:10.1371/journal.pone.0257743)
Supplement: S2 Table — (DOCX) [file pone.0257743.s003.docx]

**S2 Table.**

|  |  | **Overall** | | April | May | June | July | Aug | Sept |
| --- | --- | --- | --- | --- | --- | --- | --- | --- | --- |
| Province | |  |  |  |  |  |  |  |  |
|  | British Columbia | **1358** | **15%** | 228 (15%) | 228 (15%) | 227 (15%) | 229 (15%) | 228 (15%) | 218 (14%) |
|  | Alberta | **1862** | **21%** | 309 (21%) | 309 (21%) | 309 (21%) | 308 (21%) | 308 (21%) | 319 (21%) |
|  | Saskatchewan | **439** | **5%** | 77 (5%) | 54 (4%) | 77 (5%) | 77 (5%) | 77 (5%) | 77 (5%) |
|  | Manitoba | **496** | **6%** | 79 (5%) | 102 (7%) | 80 (5%) | 78 (5%) | 78 (5%) | 79 (5%) |
|  | Ontario | **3889** | **43%** | 648 (43%) | 647 (43%) | 648 (43%) | 649 (43%) | 649 (43%) | 648 (43%) |
|  | Atlantic | **955** | **11%** | 159 (11%) | 159 (11%) | 159 (11%) | 159 (11%) | 160 (11%) | 159 (11%) |
| Age group |  |  |  |  |  |  |  |  |  |
|  | 17-19 | **185** | **2%** | 23 (2%) | 19 (1%) | 36 (2%) | 33 (2%) | 44 (3%) | 30 (2%) |
|  | 20-29 | **1525** | **17%** | 253 (17%) | 248 (17%) | 240 (16%) | 232 (15%) | 286 (19%) | 266 (18%) |
|  | 30-39 | **1655** | **18%** | 286 (19%) | 270 (18%) | 284 (19%) | 255 (17%) | 244 (16%) | 316 (21%) |
|  | 40-49 | **1453** | **16%** | 249 (17%) | 247 (16%) | 207 (14%) | 233 (16%) | 271 (18%) | 246 (16%) |
|  | 50-59 | **1895** | **21%** | 336 (22%) | 319 (21%) | 338 (23%) | 333 (22%) | 292 (19%) | 277 (18%) |
|  | 60-69 | **1728** | **19%** | 284 (19%) | 300 (20%) | 282 (19%) | 312 (21%) | 276 (18%) | 274 (18%) |
|  | 70-79 | **518** | **6%** | 66 (4%) | 90 (6%) | 103 (7%) | 95 (6%) | 77 (5%) | 87 (6%) |
|  | 80+ | **40** | **0%** | 3 (0%) | 6 (1%) | 10 (1%) | 7 (0%) | 10 (1%) | 4 (0%) |
| Sex |  |  |  |  |  |  |  |  |  |
|  | Female | **4250** | **47%** | 719 (48%) | 699 (47%) | 669 (45%) | 725 (48%) | 728 (49%) | 710 (47%) |
|  | Male | **4749** | **53%** | 781 (52%) | 800 (53%) | 831 (55%) | 775 (52%) | 772 (51 %) | 790 (53%) |
