# Supplementary material for: Severe acute respiratory syndrome coronavirus 2 (SARS-CoV-2) seroprevalence: Navigating the absence of a gold standard
Source: PLoS One. 2021 Sep 23;16(9):e0257743. doi: 10.1371/journal.pone.0257743 (PMC8459951; doi:10.1371/journal.pone.0257743)
Supplement: S3 Table — Abbott-NP, Abbott Architect SARS-Cov-2 IgG assay targeting nucleocapsid antigen; Spike, full length spike glycoprotein; RBD, spike glycoprotein receptor binding domain; NP, nucleocapsid. (DOCX) [file pone.0257743.s004.docx]

**S3 Table .**

|  | **Non-informative Priors** | | **Informative Priors** | | **Weakly Informative Priors** | |
| --- | --- | --- | --- | --- | --- | --- |
| **Diagnostic Phenotypes** | **Observed** | **Expected** | **Observed** | **Expected** | **Observed** | **Expected** |
| **All** | 32 | 20.57 | 32 | 26.34 | 32 | 25.25 |
| **All - Abbott** | 9 | 19.31 | 9 | 18.71 | 9 | 17.78 |
| **Spike + Abbott** | 2 | 8.06 | 2 | 7.08 | 2 | 8.05 |
| **Spike + RBD** | 15 | 8.34 | 15 | 5.82 | 15 | 6.54 |
| **All – NP** | 1 | 3.28 | 1 | 3.21 | 1 | 2.61 |
| **Spike + NP** | 9 | 6.08 | 9 | 5.28 | 9 | 4.96 |
| **All – NP** | 2 | 1.62 | 2 | 1.18 | 2 | 1.22 |
| **Spike Only** | 158 | 161.85 | 158 | 161.78 | 158 | 164.69 |
| **All - Spike** | 0 | 2.51 | 0 | 1.82 | 0 | 1.92 |
| **RBD + NP** | 7 | 3.13 | 7 | 2.08 | 7 | 2.21 |
| **NP + Abbott** | 0 | 1.07 | 0 | 0.57 | 0 | 0.72 |
| **RBD Only** | 39 | 42.31 | 39 | 42.6 | 39 | 45.83 |
| **RBD + Abbott** | 0 | 0.74 | 0 | 0.54 | 0 | 0.59 |
| **NP Only** | 156 | 159.95 | 156 | 159.48 | 156 | 162.87 |
| **Abbott Only** | 17 | 18.18 | 17 | 17.15 | 17 | 20.4 |
| **None** | 8552 | 8541.99 | 8552 | 8545.35 | 8552 | 8533.35 |
| **Bayesian Information Criterion (BIC)** |  | 5372 |  | **5359** |  | 5360 |
