# Supplementary material for: Severe acute respiratory syndrome coronavirus 2 (SARS-CoV-2) seroprevalence: Navigating the absence of a gold standard
Source: PLoS One. 2021 Sep 23;16(9):e0257743. doi: 10.1371/journal.pone.0257743 (PMC8459951; doi:10.1371/journal.pone.0257743)
Supplement: S4 Table — A. Assay Characteristics Overall and Bi-monthly based on the Bayesian Latent Class Analysis with Non-Informative Priors. PPV, positive predictive value; NPV, negative predictive value; Abbott-NP, Abbott Architect SARS-Cov-2 IgG assay targeting nucleocapsid antigen; Spike, full length spike glycoprotein; RBD, spike glycoprotein receptor binding domain; NP, nucleocapsid. B. Assay Characteristics Overall and Bi-monthly based on the Bayesian Latent Class Analysis with Weakly-Informative Priors. PPV, positive predictive value; NPV, negative predictive value; Abbott-NP, Abbott Architect SARS-Cov-2 IgG assay targeting nucleocapsid antigen; Spike, full length spike glycoprotein; RBD, spike glycoprotein receptor binding domain; NP, nucleocapsid. (DOCX) [file pone.0257743.s005.docx]

**S4a Table.**

|  | **Non-Informative Priors (Overall)** | | | | **April/May** | | **June/July** | | **August/September** | |
| --- | --- | --- | --- | --- | --- | --- | --- | --- | --- | --- |
|  | Sensitivity | PPV | Specificity | NPV | Sensitivity | Specificity | Sensitivity | Specificity | Sensitivity | Specificity |
| Spike | 89.1%  (79.2, 96.9) | 28.3%  (25.9, 31.1) | 98.2%  (97.9, 98.4) | 99.9%  (99.9, 100.0) | 92.2%  (77.2, 99.4) | 97.9%  (97.4, 98.4) | 82.5%  (63.6, 96.3) | 98.8%  (98.4, 99.2) | 81.9%  (63.4, 96.2) | 97.6%  (97.0, 98.2) |
| RBD | 86.3%  (75.0, 95.3) | 60.0%  (54.8, 64.4) | 99.5%  (99.4, 99.7) | 99.9%  (99.8, 100.0) | 84.2%  (65.4, 96.7) | 99.5%  (99.2, 99.7) | 82.7%  (62.6, 97.3) | 99.6%  (99.3, 99.8) | 78.5%  (59.0, 93.9) | 99.4%  (99.0, 99.7) |
| NP | 71.8%  (60.1,82.5) | 24.2%  (22.0, 26.6) | 98.2%  (97.9, 98.4) | 99.8%  (99.7, 99.8) | 72.6%  (51.3, 90.4) | 99.5%  (99.2, 99.7) | 81.0%  (62.3, 94.7) | 97.3%  (96.7, 97.9) | 58.1%  (39.3, 76.2) | 97.6%  (97.0, 98.2) |
| Abbott-NP | 51.6%  (39.4, 64.2) | 67.9%  (64.8, 70.4) | 99.8%  (99.7, 99.9) | 99.6%  (99.5, 99.7) | 65.3%  (43.6, 85.0) | 99.7%  (99.5, 99.9) | 43.8%  (24.8, 64.6) | 99.7%  (99.5, 99.9) | 45.9%  (27.8, 65.6) | 99.9%  (99.7, 100.0) |

**S4b Table.**

|  | **Weakly-Informative Priors (Overall)** | | | | April/May | | June/July | | August/September | |
| --- | --- | --- | --- | --- | --- | --- | --- | --- | --- | --- |
|  | Sensitivity | PPV | Specificity | NPV | Sensitivity | Specificity | Sensitivity | Specificity | Sensitivity | Specificity |
| Spike | 92.9%  (83.9, 99.4) | 27.1%  (24.6, 29.8) | 98.1%  (97.8, 98.4) | 99.9%  (99.9, 100.0) | 96.7%  (87.2, 100.0) | 97.9%  (97.3, 98.4) | 92.0%  (76.9, 99.8) | 98.7%  (98.3, 99.1) | 92.2%  (77.2, 99.8) | 97.5%  (96.9, 98.0) |
| RBD | 90.6%  (80.5, 98.1) | 57.8%  (51.9, 62.5) | 99.5%  (99.3, 99.6) | 99.9%  (99.9, 100.0) | 91.3%  (77.0, 99.0) | 99.4%  (99.1, 99.7) | 93.2%  (77.9, 99.9) | 99.4%  (99.1, 99.7) | 89.7%  (73.5, 99.5) | 99.2%  (98.8, 99.5) |
| NP | 75.8%  (64.6, 85.9) | 23.0%  (20.6, 25.7) | 98.1%  (97.8, 98.4) | 99.8%  (99.8, 99.9) | 84.8%  (66.6, 97.6) | 99.4%  (99.1, 99.6) | 89.4%  (74.1, 99.3) | 97.2%  (96.6, 97.8) | 70.5%  (52.1, 88.0) | 97.5%  (96.9, 98.1) |
| Abbott-NP | 58.7%  (46.2, 71.2) | 67.2% (64.8, 70.4) | 99.8%  (99.7, 99.9) | 99.7%  (99.6, 99.8) | 79.4%  (59.8, 94.5) | 99.6%  (99.4, 99.8) | 62.9%  (42.4, 82.1) | 99.6%  (99.4, 99.8) | 63.9%  (44.1, 83.9) | 99.8%  (99.6, 99.9) |
