# Supplementary material for: Severe acute respiratory syndrome coronavirus 2 (SARS-CoV-2) seroprevalence: Navigating the absence of a gold standard
Source: PLoS One. 2021 Sep 23;16(9):e0257743. doi: 10.1371/journal.pone.0257743 (PMC8459951; doi:10.1371/journal.pone.0257743)
Supplement: S5 Table — Abbott-NP, Abbott Architect SARS-Cov-2 IgG assay targeting nucleocapsid antigen; Spike, full length spike glycoprotein; RBD, spike glycoprotein receptor binding domain; NP, nucleocapsid. (DOCX) [file pone.0257743.s006.docx]

|  |  | All | (Without Spike) | (Without RBD) | (Without NP) | (Without Abbott-NP) |
| --- | --- | --- | --- | --- | --- | --- |
| Spike | Sensitivity | 93.5% | Not included | 95.5% | 95.7% | 92.0% |
|  | Specificity | 98.1% |  | 98.1% | 98.1% | 98.2% |
| RBD | Sensitivity | 89.2% | 91.1% | Not Included | 89.8% | 88.5% |
|  | Specificity | 99.5% | 97.6% |  | 99.5% | 99.6% |
| NP | Sensitivity | 78.8% | 81.1% | 81.2% | Not Included | 78.7% |
|  | Specificity | 98.2% | 98.2% | 98.1% |  | 98.2% |
| Abbott-NP | Sensitivity | 58.5% | 62.2% | 71.1% | 62.4% | Not Included |
|  | Specificity | 99.8% | 99.8% | 99.8% | 99.8% |  |

**S5 Table.**
