# Supplementary material for: Severe acute respiratory syndrome coronavirus 2 (SARS-CoV-2) seroprevalence: Navigating the absence of a gold standard
Source: PLoS One. 2021 Sep 23;16(9):e0257743. doi: 10.1371/journal.pone.0257743 (PMC8459951; doi:10.1371/journal.pone.0257743)
Supplement: S1 Code — (DOCX) [file pone.0257743.s008.docx]

libname corr"I:\projects\covid serology_2\data";

libname analysis"I:\projects\covid serology_2\model_mtest_apr2021";

**data** prior1;

input test $ testn s s_l s_u c c_l c_u ;

cards;

spike 1 0.94 0.89 0.99 0.98 .93 1.0

RBD 2 0.89 0.84 0.95 0.99 .95 1

NP 3 0.80 0.75 0.85 0.99 .94 1

Igg 4 0.92 0.60 0.95 0.99 0.94 1

;

**run**;

**data** prior2;

input test $ testn s s_l s_u c c_l c_u ;

cards;

spike 1 0.9 0.6 1 0.95 0.9 1

RBD 2 0.9 0.6 1 0.95 0.9 1

NP 3 0.9 0.6 1 0.95 0.9 1

Igg 4 0.9 0.6 1 0.95 0.9 1

;

**proc** **print**;**run**;

**data** prior3;

input test $ testn s s_l s_u c c_l c_u ;

cards;

spike 1 0.5 0 1 0.5 0 1

RBD 2 0.5 0 1 0.5 0 1

NP 3 0.5 0 1 0.5 0 1

Igg 4 0.5 0 1 0.5 0 1

;

**proc** **print**;**run**;

**%macro** trans(fn);

data t&fn;set beta;

if testn=&fn;

s&fn=s;c&fn=c; beta_c&fn=beta_c;alpha_c&fn=alpha_c ;beta_s&fn =beta_s; alpha_s&fn=alpha_s;

keep beta_c&fn alpha_c&fn beta_s&fn alpha_s&fn;

run;

proc print;run;

**%mend**;

%let prior=prior1;

%let testresult=Test_result_monthall4_9_06apr21;

%let post=Post_4test_all_p1;

**%macro** mcmc(prior,testresult,post);

data beta; set &prior;

c_v=((c_u-c_l)/(**4**))****2**;

alpha_c=(**1**-c)*c*c/c_v-c;

beta_c=alpha_c*(**1**-c)/c;

s_v=((s_u-s_l)/(**4**))****2**;

alpha_s=(**1**-s)*s*s/s_v-s;

beta_s=alpha_s*(**1**-s)/s;

keep test testn c s beta_c alpha_c beta_s alpha_s;

run;

%***trans***(**1**);%***trans***(**2**); %***trans***(**3**); %***trans***(**4**)

data beta3;merge t1 t2 t3 t4 analysis.&testresult;run;

proc mcmc data=beta3 seed=**123** outpost=analysis.&post._&sysdate

diag=ess nbi=**5000** nthin=**50** nmc=**5000000** statistics=(summary interval);

alpha_phi=**1**; beta_phi=**1**;

parms phi **0.02**

s1 **0.94** c1 **0.99** s2 **0.92** c2 **0.89**

s3 **0.9** c3 **0.95** s4 **0.9** c4 **0.96**

Y1 **5** Y2 **1** Y3 **0** Y4 **1** Y5 **0** Y6 **1** Y7 **0** Y8 **1**

Y9 **0** Y10 **0** Y11 **0** Y12 **1** Y13 **0** Y14 **1** Y15 **1** Y16 **1** ;

p1= phi*S1*S2*S3*S4 /( phi*S1*S2*S3*S4 + (**1**-phi)*(**1**-C1)*(**1**-C2)*(**1**-C3)*(**1**-C4) );

p2= phi*S1*S2*S3*(**1**-S4) /( phi*S1*S2*S3*(**1**-S4) + (**1**-phi)*(**1**-C1)*(**1**-C2)*(**1**-C3)*C4 );

p3= phi*S1*S2*(**1**-S3)*S4 /( phi*S1*S2*(**1**-S3)*S4 + (**1**-phi)*(**1**-C1)*(**1**-C2)*C3*(**1**-C4) );

p4= phi*S1*S2*(**1**-S3)*(**1**-S4) /( phi*S1*S2*(**1**-S3)*(**1**-S4) + (**1**-phi)*(**1**-C1)*(**1**-C2)*C3*C4 );

p5= phi*S1*(**1**-S2)*S3*S4 /( phi*S1*(**1**-S2)*S3*S4 + (**1**-phi)*(**1**-C1)*C2*(**1**-C3)*(**1**-C4) );

p6= phi*S1*(**1**-S2)*S3*(**1**-S4) /( phi*S1*(**1**-S2)*S3*(**1**-S4) + (**1**-phi)*(**1**-C1)*C2*(**1**-C3)*C4 );

p7= phi*S1*(**1**-S2)*(**1**-S3)*S4 /( phi*S1*(**1**-S2)*(**1**-S3)*S4 + (**1**-phi)*(**1**-C1)*C2*C3*(**1**-C4) );

p8= phi*S1*(**1**-S2)*(**1**-S3)*(**1**-S4) /( phi*S1*(**1**-S2)*(**1**-S3)*(**1**-S4) + (**1**-phi)*(**1**-C1)*C2*C3*C4 );

p9= phi*(**1**-S1)*S2*S3*S4 /( phi*(**1**-S1)*S2*S3*S4 + (**1**-phi)*C1*(**1**-C2)*(**1**-C3)*(**1**-C4) );

p10= phi*(**1**-S1)*S2*S3*(**1**-S4) /( phi*(**1**-S1)*S2*S3*(**1**-S4) + (**1**-phi)*C1*(**1**-C2)*(**1**-C3)*C4 );

p11= phi*(**1**-S1)*S2*(**1**-S3)*S4 /( phi*(**1**-S1)*S2*(**1**-S3)*S4 + (**1**-phi)*C1*(**1**-C2)*C3*(**1**-C4) );

p12= phi*(**1**-S1)*S2*(**1**-S3)*(**1**-S4) /( phi*(**1**-S1)*S2*(**1**-S3)*(**1**-S4) + (**1**-phi)*C1*(**1**-C2)*C3*C4 );

p13= phi*(**1**-S1)*(**1**-S2)*S3*S4 /( phi*(**1**-S1)*(**1**-S2)*S3*S4 + (**1**-phi)*C1*C2*(**1**-C3)*(**1**-C4) );

p14= phi*(**1**-S1)*(**1**-S2)*S3*(**1**-S4) /( phi*(**1**-S1)*(**1**-S2)*S3*(**1**-S4) + (**1**-phi)*C1*C2*(**1**-C3)*C4 );

p15= phi*(**1**-S1)*(**1**-S2)*(**1**-S3)*S4 /( phi*(**1**-S1)*(**1**-S2)*(**1**-S3)*S4 + (**1**-phi)*C1*C2*C3*(**1**-C4) );

p16= phi*(**1**-S1)*(**1**-S2)*(**1**-S3)*(**1**-S4) /( phi*(**1**-S1)*(**1**-S2)*(**1**-S3)*(**1**-S4) + (**1**-phi)*C1*C2*C3*C4 );

;

prior Y1 ~ binomial(a1, p1);

prior Y2 ~ binomial(a2, p2);

prior Y3 ~ binomial(a3, p3);

prior Y4 ~ binomial(a4, p4);

prior Y5 ~ binomial(a5, p5);

prior Y6 ~ binomial(a6, p6);

prior Y7 ~ binomial(a7, p7);

prior Y8 ~ binomial(a8, p8);

prior Y9 ~ binomial(a9, p9);

prior Y10 ~ binomial(a10, p10);

prior Y11 ~ binomial(a11, p11);

prior Y12 ~ binomial(a12, p12);

prior Y13 ~ binomial(a13, p13);

prior Y14 ~ binomial(a14, p14);

prior Y15 ~ binomial(a15, p15);

prior Y16 ~ binomial(a16, p16);

YYtotal=sum(Y1,Y2,Y3,Y4,Y5,Y6,Y7,Y8,Y9,Y10,Y11,Y12,Y13,Y14,Y15,Y16);

YY11=sum(Y1,Y2,Y3,Y4,Y5,Y6,Y7,Y8);

YY12=YYtotal-YY11;

YY21=sum(Y1,Y2,Y3,Y4,Y9,Y10,Y11,Y12);

YY22=YYtotal-YY21;

YY31=sum(Y1,Y2,Y5,Y6,Y9,Y10,Y13,Y14);

YY32=YYtotal-YY31;

YY41=sum(Y1,Y3,Y5,Y7,Y9,Y11,Y13,Y15);

YY42=YYtotal-YY41;

****AA****;

N=sum(A1,A2,A3,A4,A5,A6,A7,A8,A9,A10,A11,A12,A13,A14,A15,A16);

AA11=sum(A1,A2,A3,A4,A5,A6,A7,A8);

AA12=N-AA11;

AA21=sum(A1,A2,A3,A4,A9,A10,A11,A12);

AA22=N-AA21;

AA31=sum(A1,A2,A5,A6,A9,A10,A13,A14);

AA32=N-AA31;

AA41=sum(A1,A3,A5,A7,A9,A11,A13,A15);

AA42=N-AA41;

prior phi~ beta(YYtotal+ alpha_phi,N-YYtotal+beta_phi);

prior S1 ~ beta(YY11+alpha_s1,YY12+beta_s1);

prior C1 ~ beta(AA12-YY12+alpha_c1,AA11-YY11+beta_c1);

prior S2 ~ beta(YY21+alpha_s2,YY22+beta_s2);

prior C2 ~ beta(AA22-(YY22)+alpha_c2,AA21-(YY21)+beta_c2);

prior S3 ~ beta(YY31+alpha_s3,YY32+beta_s3);

prior C3 ~ beta(AA32-(YY32)+alpha_c3,AA31-(YY31)+beta_c3);

prior S4 ~ beta(YY41+alpha_s4,YY42+beta_s4);

prior C4~ beta(AA42-(YY42)+alpha_c4,AA41-(YY41)+beta_c4);

model general(**0**);

run;

**%mend**;

%***mcmc***(prior1, monthall4_9_26apr21,Post_4test_all_prior1);

%***mcmc***(prior2, monthall4_9_26apr21,Post_4test_all_prior2);

%***mcmc***(prior3, monthall4_9_26apr21,Post_4test_all_prior3);

%***mcmc***(prior1, month4_5_26apr21,Post_4test_4_5_prior1);

%***mcmc***(prior2, month4_5_26apr21,Post_4test_4_5_prior2);

%***mcmc***(prior3, month4_5_26apr21,Post_4test_4_5_prior3);

%***mcmc***(prior1, month6_7_26apr21,Post_4test_6_7_prior1);

%***mcmc***(prior2, month6_7_26apr21,Post_4test_6_7_prior2);

%***mcmc***(prior3, month6_7_26apr21,Post_4test_6_7_prior3);

%***mcmc***(prior1, month8_9_26apr21,Post_4test_8_9_prior1);

%***mcmc***(prior2, month8_9_26apr21,Post_4test_8_9_prior2);

%***mcmc***(prior3, month8_9_26apr21,Post_4test_8_9_prior3);

Import Data:

|  | **A1** | **A2** | **A3** | **A4** | **A5** | **A6** | **A7** | **A8** | **A9** | **A10** | **A11** | **A12** | **A13** | **A14** | **A15** | **A16** | **Data file :** |
| --- | --- | --- | --- | --- | --- | --- | --- | --- | --- | --- | --- | --- | --- | --- | --- | --- | --- |
| **All 4-9** | 32 | 9 | 2 | 15 | 1 | 9 | 2 | 158 | 0 | 7 | 0 | 39 | 0 | 156 | 17 | 8552 | monthall4_9_26apr21 |
| **Month 4-5** | 11 | 2 | 1 | 4 | 1 | 1 | 0 | 59 | 0 | 0 | 0 | 12 | 0 | 14 | 8 | 2886 | month4_5_26apr21 |
| **Month 6-7** | 10 | 5 | 0 | 3 | 0 | 4 | 0 | 32 | 0 | 3 | 0 | 11 | 0 | 76 | 7 | 2849 | month6_7_26apr21 |
| **Month 8-9** | 11 | 2 | 1 | 8 | 0 | 4 | 2 | 67 | 0 | 4 | 0 | 16 | 0 | 66 | 2 | 2817 | month8_9_26apr21 |

Calculate estimates:

proc means mean median q1 q3 data= ANALYSIS.Post_4test_all_prior1_26apr21;

var phi s1 c1 s2 c2 s3 c3 s4 c4;

run;

proc means mean median q1 q3 data= ANALYSIS.Post_4test_all_prior2_26apr21;

var phi s1 c1 s2 c2 s3 c3 s4 c4;

run;

proc means mean median q1 q3 data= ANALYSIS.Post_4test_all_prior3_26apr21;

var phi s1 c1 s2 c2 s3 c3 s4 c4;

run;

…..
